# Implementation of a tuberculosis elimination project, India 2018–2019

**DOI:** 10.2471/BLT.22.288277

**Published:** 2023-01-18

**Authors:** Aakshi Kalra, Akhil S ThekkePurakkal, Raghuram Rao, Debadutta Parija, Vaibhav Ghule, Ajaz Lone, Tajamul Showket, Rajendra P Joshi, Sanjay Sarin, Sarabjit S Chadha

**Affiliations:** a FIND, Flat No. 8, 9th Floor, Vijaya Building, 17 Barakhamba Road, New Delhi 110001, India.; b Central TB Division, Ministry of Health and Family Welfare, New Delhi, India.; c FIND, Geneva, Switzerland.

## Abstract

**Objective:**

To describe the changes in tuberculosis case notifications by the private sector after implementation of the Joint Effort for Elimination of Tuberculosis project in India in 2018.

**Methods:**

We retrieved data from the project recorded in India’s national tuberculosis surveillance system. We analysed data on 95 project districts in six states (Andhra Pradesh, Himachal Pradesh, Karnataka, Punjab including Chandigarh, Telangana and West Bengal) to assess changes in the number of tuberculosis notifications, private provider notifiers and microbiological confirmations of cases from 2017 (baseline) to 2019. We compared case notification rates in districts where the project was implemented with the rates in districts where it was not.

**Findings:**

From 2017 to 2019, tuberculosis notifications increased by 138.1% (from 44 695 to 106 404), and case notification rates more than doubled from 20 to 44 per 100 000 population. The number of private notifiers increased by over threefold, from 2912 to 9525, during this period. The number of microbiologically confirmed pulmonary and extra-pulmonary tuberculosis cases notified increased by more than two times (from 10 780 to 25 384) and nearly three times (from 1477 to 4096), respectively. The districts where the project was implemented showed a 150.3% increase in case notification rates per 100 000 population from 2017 to 2019 (from 16.8 to 41.9) while in non-project districts, this increase was only 89.8% (from 6.1 to 11.6).

**Conclusion:**

The substantial increase in tuberculosis notifications demonstrate the value of the project in engaging the private sector. Scaling up these interventions is important to consolidate and extend these gains towards tuberculosis elimination.

## Résumé

**Objectif:**

Décrire les changements opérés en matière de signalement des cas de tuberculose par le secteur privé après le déploiement du projet Joint Effort for Elimination of Tuberculosis en Inde en 2018.

**Méthodes:**

Nous avons extrait les données du projet enregistré dans le système national de surveillance de la tuberculose en Inde. Nous avons ensuite analysé les informations relatives aux 95 districts repris dans le projet, répartis sur six États (Andhra Pradesh, Himachal Pradesh, Karnataka, Punjab – Chandigarh inclus –, Télangana et Bengale-Occidental), afin de mesurer l'évolution au niveau du nombre de signalements de cas de tuberculose, des auteurs de signalements parmi les prestataires privés, et de la confirmation microbiologique des cas entre 2017 (période initiale) et 2019. Nous avons comparé le taux de signalement entre les districts où le projet avait été mis en œuvre et ceux où il ne l'avait pas été.

**Résultats:**

Entre 2017 et 2019, les cas de tuberculose signalés ont augmenté de 138,1% (de 44 695 à 106 404), tandis que le taux de signalement a plus que doublé, passant de 20 à 44 pour 100 000 habitants. Durant cette période, le nombre d'auteurs de signalements issus du privé a été multiplié par trois, passant de 2912 à 9525. Le nombre de cas de tuberculose ayant fait l'objet d'une confirmation microbiologique a plus que doublé (de 10 780 à 25 384) pour la tuberculose pulmonaire et presque triplé (de 1477 à 4096) pour la tuberculose extrapulmonaire. Les districts où le projet a été mis en œuvre ont connu une hausse de 150,3% en termes de signalement des cas par 100 000 habitants entre 2017 et 2019 (de 16,8 à 41,9), contre seulement 89,8% (de 6,1 à 11,6) dans les districts non concernés par le projet.

**Conclusion:**

La nette progression du nombre de signalements de tuberculose montre qu'un tel projet favorise l'implication du secteur privé. Multiplier ce type d'action est essentiel pour développer et renforcer son rôle dans l'élimination de la tuberculose.

## Resumen

**Objetivo:**

Describir los cambios en las notificaciones de casos de tuberculosis que realiza el sector privado tras la ejecución del proyecto Joint Effort for Elimination of Tuberculosis (Esfuerzo conjunto para la eliminación de la tuberculosis) en la India en 2018.

**Métodos:**

Se recuperaron datos del proyecto registrados en el sistema nacional de vigilancia de la tuberculosis de la India. Se analizaron los datos de 95 distritos del proyecto en seis estados (Andhra Pradesh, Himachal Pradesh, Karnataka, Punjab, incluido Chandigarh, Telangana y Bengala Occidental) para evaluar los cambios en la cantidad de notificaciones de tuberculosis, los notificadores de proveedores privados y las confirmaciones microbiológicas de casos desde 2017 (base de referencia) hasta 2019. Se compararon las tasas de notificación de casos en los distritos donde se ejecutó el proyecto con las tasas en los distritos donde no se ejecutó.

**Resultados:**

De 2017 a 2019, las notificaciones de tuberculosis aumentaron un 138,1 % (de 44 695 a 106 404), y las tasas de notificación de casos aumentaron más del doble, de 20 a 44 por cada 100 000 habitantes. La cantidad de notificadores privados se multiplicó por más de tres, de 2912 a 9525, durante este periodo. La cantidad de casos notificados de tuberculosis pulmonar y extrapulmonar confirmados por pruebas microbiológicas aumentó más del doble (de 10 780 a 25 384) y casi el triple (de 1477 a 4096), respectivamente. Los distritos donde se ejecutó el proyecto mostraron un aumento del 150,3 % en las tasas de notificación de casos por cada 100 000 habitantes de 2017 a 2019 (de 16,8 a 41,9), mientras que en los distritos donde no se ejecutó el proyecto, este aumento fue solo del 89,8 % (de 6,1 a 11,6).

**Conclusión:**

El aumento sustancial de las notificaciones de tuberculosis demuestra el valor del proyecto al implicar al sector privado. La ampliación de estas intervenciones es importante para consolidar y prolongar estos avances hacia la eliminación de la tuberculosis.

## ملخص

**الغرض:**

وصف التغييرات في بلاغات حالات الإصابة بمرض السل من جانب القطاع الخاص بعد تنفيذ مشروع الجهود المشتركة للقضاء على مرض السل في الهند عام 2018.

**الطريقة:**

قمنا باسترجاع البيانات من المشروع المسجل في النظام الوطني لمراقبة مرض السل في الهند. قمنا بتحليل البيانات الخاصة بـ 95 منطقة للمشروع في ست ولايات (أندرا براديش، وهيماشال براديش، وكارناتاكا، والبنجاب بما في ذلك شانديجاره، وتيلانجانا، والبنجال الغربية) لتقييم التغييرات في عدد بلاغات الإصابة بالسل، وبلاغات مقدمي الخدمات الخاصين، والتأكيدات الميكروبيولوجية للحالات اعتبارًا من عام 2017 (خط الأساس) إلى عام 2019. قمنا بمقارنة معدلات الإبلاغ عن الحالات في المناطق التي تم تنفيذ المشروع فيها، مع المعدلات في المناطق التي لم يتم فيها التنفيذ.

**النتائج:**

من عام 2017 إلى عام 2019، زادت بلاغات الإصابة بمرض السل بنسبة %138.1 (من 44695 إلى 106404)، وتضاعفت معدلات الإبلاغ عن الحالات لأكثر من الضعف من 20 إلى 44 لكل 100000 من السكان. زادت بلاغات مقدمي الخدمة الخاصين لأكثر من ثلاثة أضعاف، من 2912 إلى 9525، خلال هذه الفترة. زاد عدد الحالات المؤكدة ميكروبيولوجيًا للسل الرئوي والسل خارج الرئة التي تم الإبلاغ عنها بأكثر من مرتين (من 10780 إلى 25384)، وما يقرب من ثلاث مرات (من 1477 إلى 4096)، على التوالي. أظهرت المناطق التي تم تنفيذ المشروع فيها زيادة بنسبة %150.3 في معدلات الإبلاغ عن الحالات لكل 100000 نسمة من عام 2017 إلى عام 2019 (من 16.8 إلى 41.9)، بينما كانت هذه الزيادة في المناطق غير التابعة للمشروع 89.8% فقط (من 6.1 إلى 11.6).

**الاستنتاج:**

إن الزيادة الملموسة في البلاغات عن الإصابة بالسل، تشير إلى قيمة المشروع في إشراك القطاع الخاص. يعد توسيع نطاق هذه التدخلات أمرًا مهمًا لتدعيم وزيادة هذه المكاسب نحو القضاء على السل.

## 摘要

**目的:**

旨在描述 2018 年印度实施《共同努力消除结核病》项目之后私营部门结核病病例通报方面发生的改变。

**方法:**

我们检索了印度国家结核病监测系统中记录项目的数据。我们分析了分布在六个邦【安得拉邦、卡纳塔克邦、旁遮普邦（包括昌迪加尔）、特伦甘纳邦和喜马偕尔邦、西孟加拉邦】的 95 个项目地区的数据，以评估 2017 年（基线）至 2019 年结核病通报、私营机构通告和微生物学鉴定数量的变化。我们比较了实施项目地区和未实施项目地区的病例通报率。

**结果:**

从 2017 年到 2019 年，结核病通报数量增加了 138.1%（从 44,695 例增加到 106,404 例）；病例通报率增加了不止一倍，从每 100,000 人 20 例增加到 44 例。同一时期，私营部门通告的数量增加了超过三倍，从 2912 例增至 9525 例。肺部微生物鉴定的数量增加了不止两倍（从 10,780 例增至 25,384 例）；肺外器官结核病病例通报的数量增加了约三倍（从 1,477 例增至 4,096 例）。数据显示，从 2017 年到 2019 年，实施项目地区每 100,000 人的病例通报率增加了 150.3%（从 16.8 例增至 41.9 例），而在未实施项目的地区，病例通报率仅增加了 89.8%（从 6.1 例增至 11.6 例）。

**结论:**

结核病通报数量的大幅增长表明该项目对调动私营部门参与具有重大价值。大规模实施此类干预对巩固和扩大在消除结核病方面所取得的收益非常重要。

## Резюме

**Цель:**

Описать изменения в уведомлениях о случаях заболевания туберкулезом в частном секторе после реализации проекта «Совместные усилия по ликвидации туберкулеза» в Индии в 2018 году.

**Методы:**

Были получены данные проекта, зарегистрированные в индийской национальной системе надзора за туберкулезом. В рамках проекта были изучены данные по 95 округам в шести штатах (Андхра-Прадеш, Карнатака, Пенджаб, Химачал-Прадеш, в том числе Западная Бенгалия, Телангана и Чандигарх) для оценки изменений в количестве уведомлений о заболевании туберкулезом, уведомлений от поставщиков услуг частных учреждений и подтверждений случаев заболевания по результатам микробиологических исследований с 2017 (исходный уровень) по 2019 год. Сравнивались показатели регистрируемой заболеваемости в округах, где проект был реализован, с показателями в округах, на территории которых он не был реализован.

**Результаты:**

С 2017 по 2019 год количество уведомлений о заболевании туберкулезом увеличилось на 138,1% (с 44 695 до 106 404), показатели регистрируемой заболеваемости выросли более чем в два раза: с 20 до 44 на 100 000 жителей. За этот период число частных уведомителей увеличилось более чем в три раза (с 2912 до 9525). Количество зарегистрированных случаев заболевания легочным и внелегочным туберкулезом по результатам микробиологических исследований увеличилось более чем в два раза (с 10 780 до 25 384) и почти в три раза (с 1477 до 4096) соответственно. В округах, где был реализован проект, показатель регистрируемой заболеваемости на 100 000 жителей с 2017 по 2019 год увеличился на 150,3% (с 16,8 до 41,9), тогда как в не охваченных проектом округах этот показатель составил всего 89,8% (с 6,1 до 11,6).

**Вывод:**

Значительное увеличение количества уведомлений о заболевании туберкулезом указывает на ценность проекта в плане привлечения частного сектора. Для закрепления и расширения этих достижений на пути к ликвидации туберкулеза важно расширить масштабы проведения подобных мероприятий.

## Introduction

India, which accounts for more than a quarter of the global tuberculosis burden, aims to eliminate tuberculosis in the country by 2025.^1^^,^^2^ Engagement of the private sector is crucial to achieve this goal. Nearly three quarters of tuberculosis patients in India still approach the unregulated private sector as their first point of health care, and almost half of the patients take treatment from private health-care providers.^3^^–^^7^ India’s national tuberculosis programme has introduced and supported several public–private mix initiatives since the late 1990s. However, the National Strategic Plan, 2017–2025 for tuberculosis control highlighted inefficiencies in the scale of private sector engagement in such initiatives despite the large role the private sector plays in tuberculosis care in India.^2^^,^^8^^,^^9^ Low case notification, especially from the private sector, has been a major impediment in ensuring good quality care for tuberculosis patients.

After tuberculosis was made a notifiable disease in India in 2012,^10^ *Ni-kshay*, a web-based patient management system, was launched to strengthen tuberculosis surveillance in the country. Through the national programme’s strategic guidance, many private health-care providers were made aware of the necessity for and process of tuberculosis case notification.^11^^–^^15^ Consequently, the private sector’s contribution to tuberculosis case notifications increased from less than 1% (3547/1 467 585) in 2012 to 21.0% (383 784/1 827 959) in 2017.^3^^,^^11^^,^^16^ Despite this increase, about a million cases were not notified in 2017.^16^

The Global Fund to Fight AIDS, Tuberculosis and Malaria has supported the Joint Effort for Elimination of Tuberculosis project, which has been implemented in 23 states in India since 2018. This project is the largest concerted effort to engage the private sector in the country. The project is being implemented by FIND, the global alliance for diagnostics, and two consortium partners in close collaboration with implementing agencies, the national tuberculosis programme, state and district tuberculosis offices, the World Health Organization (WHO) and other stakeholders. The key objectives of the project are to ensure and enhance notification of tuberculosis cases and improve tuberculosis treatment success rates through engagement of the private sector.

Of all tuberculosis cases notified in India in 2019, 28.2% (678 895/2 404 815) were from the private sector.^13^ The gap between the estimated incidence of tuberculosis and notified cases fell by 74.9%, from 900 000 cases in 2017 to 235 000 cases in 2019. Despite the widespread presence of the project since 2018, its contribution to this significant decline has not been documented.

In this paper, we aim to describe the findings from implementation of the project in areas covered by FIND from 2017 to 2019, by comparing: (i) tuberculosis case notifications; (ii) private providers engaged; and (iii) microbiologically confirmed cases among cases notified. We also compared tuberculosis case notification rates in districts where the project was implemented with rates in districts where it was not.

## Methods

### Study design

We conducted a cross-sectional study which analysed routine programme data.

### Setting

The Joint Effort for Elimination of Tuberculosis project was implemented by FIND in six states in India: Andhra Pradesh, Himachal Pradesh, Karnataka, Punjab (including Chandigarh), Telangana and West Bengal from 2018 onwards. By the beginning of 2019, the project had started in 95 of 146 districts in these states, covering a population of 245 million.

### Project implementation

The private provider engagement under the project was implemented through a human resource-intensive patient-provider support agency model, and a human resource-limited patient-provider support agency lite model.^17^ The human resource-intensive model included senior field officers, field officers, hub agents, sample collection and transportation agents, and treatment coordinators. The resource-limited patient-provider support agency lite model had only one primary dedicated human resource (Box 1 for operational definitions).

Box 1 **Operational definitions of variables in the Joint Effort for Elimination of Tuberculosis project, India**

**Intervention models and implementation variables**

1. Patient-provider support agency model/district.

A human resource-intensive patient-provider support agency model in the Joint Effort for Elimination of Tuberculosis project. The main human resources included senior field officers, field officers, hub agents, sample collection and transportation agents, and treatment coordinators. The model focused on provider engagement and patient services. In this model, implementation was done through an intermediary agency at the ground level. These agencies reported on tuberculosis case notifications and treatment outcomes including other qualitative indicators, such as microbiological confirmation, enrolment delays and adherence percentage, with support of the field team. The districts were those in the national tuberculosis programme where the patient-provider support agency model was implemented.

1. Services facilitated by the resource-intensive patient-provider support agency model.

- Linking to free cartridge-based nucleic acid amplification assay test diagnostics;
- Providing free sample collection and transportation for diagnosis;
- Linking diagnosed tuberculosis patients to free treatment provided under the national tuberculosis programme;
- Ensuring adherence to treatment through active engagement with patients and providers through regular follow-up calls, visits and reminders; and
- Facilitating provision of incentives to providers on successful notification and treatment outcome, and to patients for supporting their treatment through the national tuberculosis programme’s *Ni-kshay Poshan Yojna*.^2^^,^^18^

1. Patient-provider support agency lite model/district.

A patient-provider support agency model in the Joint Effort for Elimination of Tuberculosis project implemented using fewer human resources. The model focused on private-provider engagement through strengthening capacity of the national tuberculosis programme staff and was directly implemented by FIND (no intermediary agencies were involved). This model has only one primary dedicated human resource (city officer). This resource catered to 3–4 districts and supported the national tuberculosis programme staff in the following public–private integration activities.

- Identifying providers catering to a large number of tuberculosis patients in each district;
- Engaging providers catering to a large number of tuberculosis patients, pharmacies and laboratories in the district through one-on-one visits and continuing medical education programmes to: raise awareness of the project activities and the role of the private sector in case notifications; and promote the use of cartridge-based nucleic acid amplification tests and tuberculosis treatment methods available in the public sector;
- Recording tuberculosis notifications and treatment outcomes in *Ni-kshay*;
- Linking patients to free diagnostics, treatment, support for adherence to treatment and incentives provided by the national tuberculosis programme;
- Providing technical assistance to extend patient links to social welfare schemes; and
- Strengthening the capacity of the national tuberculosis programme staff including private–public coordinators, tuberculosis health visitors and tuberculosis treatment supervisors in private–public activities to enhance engagement with private providers.

Reporting of notifications and treatment outcomes was done by the national tuberculosis programme staff with support of project field staff which was coordinated by the state project lead. The districts in the national tuberculosis programme where the patient-provider support agency lite model was implemented were referred to as patient-provider support agency lite districts.

1. Hub

Refers to a prominent private health facility in the resource-intensive patient-provider support agency district which catered to a large number of tuberculosis patients (Box 2).

1. Spoke

Refers to private facilities engaged by the project, such as clinics and stand-alone practitioners, among others, which were linked to a hub for project services (Box 2).

**Tuberculosis, Ni-kshay and analysis variables**

1. Tuberculosis case

Patient diagnosed with at least one clinical specimen positive for acid-fast bacilli or culture-positive for *Mycobacterium tuberculosis* or positive for tuberculosis using a rapid diagnostic molecular test, or diagnosed clinically as a case of tuberculosis without microbiological confirmation and started on antituberculosis drugs.

1. Notified tuberculosis case

New cases, recurrent cases, patients returning after treatment interruption or put on new treatment regimen due to failure of current treatment regimen, pulmonary or extra-pulmonary tuberculosis cases reported to the *Ni-kshay* registry. Cases can be either rifampicin-resistant, rifampicin-sensitive or with unknown drug sensitivity.^10^

1. Provider

Private practitioner or clinic (single) and hospital, clinic, nursing home (multi), pharmacies and laboratories registered in *Ni-kshay* for tuberculosis case notification.

1. Notifier

Registered private-sector provider who has notified, enrolled or referred at least one tuberculosis-positive case to *Ni-kshay* in the year.

1. Microbiological cases

Tuberculosis cases diagnosed using cartridge-based nucleic acid amplification assay test, culture, smear microscopy, first- and second-line probe assays, or Truenat (MTB/MTB-RIF) tests.

1. Clinical cases

Tuberculosis cases diagnosed using chest X-ray and methods of diagnosis other than microbiological methods.

MTB: *Mycobacterium tuberculosis*; RIF: resistance to rifampicin.

The human resource-intensive patient-provider support agency model was implemented in 21 districts through three intermediary agencies. In this model, private providers in each district at all touch points (namely, stand-alone clinics, hospitals, laboratories and pharmacies) were systematically mapped using data from various sources (available in the online repository)^19^ including other projects, such as the paediatric tuberculosis project.^15^ The project’s field staff confirmed this information at the state level through personal visits to the facilities. Details of the project implementation are provided in Box 2. The link between private provider facilities was optimized using a hub and spoke model (Box 1 and Box 2).^15^ Hubs were prominent facilities in the private sector in a district where several tuberculosis patients sought care. The hubs facilitated project services including enrolment of presumptive tuberculosis patients, sample collection and notification of confirmed cases through *Ni-kshay*. The spokes were small private hospitals, clinics, stand-alone practitioners, among others, which were linked to a hub for project services including diagnosis and treatment. The project staff used planned follow-up visits and electronic and telephone communications to ensure active engagement of the enrolled providers throughout the process (Box 2). The patient-provider support agency lite model was implemented in 74 districts and focused on engaging private providers through capacity-building of the national tuberculosis programme staff (Box 1).

Box 2 **Key implementation activities in the resource-intensive patient-provider support agency model**

**Provider mapping and sensitization**

In each state, the team lead at the state level and field officers performed the provider mapping exercise (available in the online repository).^19^ Data on providers, including case load of presumptive tuberculosis patients per month, historic trends in tuberculosis case notification and number of samples referred for diagnosis of tuberculosis, were collected through the mapping exercise. Based on the distribution of the case load of presumptive tuberculosis patients, providers were categorized into: (i) category A – providers attending to ≥ 30 cases a month; (ii) category B – providers attending to 11 to 29 cases a month; and (iii) category C – providers attending to 1 to 10 cases a month. This categorization was performed to optimize the visits to providers given the limited field staff available (2–3 field officers per district), and to ensure a consistent flow of notifications from high notifiers and improved notifications from low notifiers. Accordingly, providers in categories A, B and C were visited three, two and one time per month, respectively, to highlight the importance of notification, and update them on the latest guidelines on tuberculosis management and the support provided by the project for diagnosis, notification and treatment adherence of tuberculosis patients. These visits were scheduled for the convenience of the providers and could be within or outside regular working hours of the providers, or at weekends.

Apart from one-on-one visits, other strategies were used to maintain the active engagement of the providers such as: group meetings for continuing medical education sessions; peer-to-peer influence; information and education materials; telephonic follow-up; and engagement of professional bodies of providers. Between 2018 and 2019, 100 continuing medical education sessions were conducted, and each session was attended by 50 providers on average. Selected providers who could encourage fellow providers and bring about a positive change in tuberculosis case notifications among peers were engaged for peer-to-peer sensitization. The project staff also used the regular meetings of professional bodies of providers to raise awareness of the project.

**Hub and spoke model**

In the resource-intensive patient-provider support agency model, hubs were prominent facilities in the private sector in a district where several tuberculosis patients seek care. The criteria for a facility to qualify as a hub were: (i) acceptance by an appropriate authority (for example, head or administrator of the facility) to be a hub facility (verbal or written); (ii) acceptance of tuberculosis notification practices as per guidelines on standards of tuberculosis care in India by private providers or doctors of the facility; (iii) available seating for project field staff (hub agent; available in the online repository);^19^ (iv) availability of spoke facilities nearby for diagnostic and/or treatment referrals base on the project mapping data; and (v) access to linked tuberculosis diagnostic laboratories within the project setting at a distance of no more than 25 km or travel time of no more than 2 hours one way. Spokes were private facilities engaged by the project (small hospitals, clinics and individual practitioners, among others) which were linked to a hub for project services including diagnosis, treatment and adherence support. In all, 255 hubs and 13 566 spokes were established during the project in all 21 patient-provider support agency districts.

**Sample collection and transportation, report delivery and patient notification**

Once a facility was established as hub, a hub agent was placed at the facility to coordinate with providers and patients for sample collection and referral (available in the online repository),^19^ and to support enrolment of presumptive tuberculosis patients in *Ni-kshay* with the relevant documentation. At a spoke, samples were either directly collected from presumptive tuberculosis patients or the patients were referred to the hub facility. At both hubs and spokes, a designated agent supported the collection and transportation of samples to the assigned diagnostic facility, and the collection of reports and their delivery to hubs and spokes in coordination with the hub agent (available in the online repository).^19^ Diagnosed patients were notified in *Ni-kshay* by the hub agent or directly by the providers.

**Link to treatment and follow-up**

Once a tuberculosis case was notified in *Ni-kshay*, the doctor or hub agent linked the patient to appropriate treatment and assigned a dedicated field staff (treatment coordinator) to the patient. The treatment coordinator provided treatment and adherence support to patients through face-to-face interactions, regular follow-ups (weekly in the intensive phase and fortnightly in the continuation phase of treatment), monthly drug refill reminders, and drug delivery depending on the need of the patient. In addition, the treatment coordinator also facilitated patients’ links to the public sector for treatment, government schemes for nutrition and motivation, and recording in *Ni-kshay*.

The cost of implementation of the project for 3 years was budgeted at 6 332 602 United States dollars.

### Cases included

We included in the analysis all tuberculosis cases notified in *Ni-kshay* through the project between January 2018 and December 2019. We also included cases notified in 2017 from the project areas.

### Data source and variables

We used data drawn from *Ni-kshay* version 2.0, which captured data from 2017 and the project years (2018–2019) as part of routine monitoring.

We extracted anonymized data from *Ni-kshay* on providers and patients including the following information: individual patient data on age, gender (female, male and third gender), site of tuberculosis (pulmonary or extra-pulmonary), basis of diagnosis (microbiological or clinical; Box 2), human immunodeficiency virus (HIV) status (positive or negative), facility enrolled in (state and district) and date of enrolment. We compared the 2019 data with data from 2017, the year before the start of the project, to determine the change in the parameters related to the study objective, namely changes in: the number of tuberculosis cases notified; the case notification rate per 100 000 population; the number of private-sector providers that notified, enrolled or referred at least one tuberculosis-positive case in *Ni-kshay* in the year; and the basis of diagnosis, microbiological or clinical. We also compared trends in notification rates between project and non-project districts in states where not all districts were covered by the project, namely Karnataka, Punjab, West Bengal, Telangana and Himachal Pradesh. Andhra Pradesh and Chandigarh were not included because all districts were covered by the project.

The field staff recorded the data on patients and providers in structured daily diaries and registers. The state programme management units maintained these records. All project data were entered in *Ni-kshay* as part of routine monitoring. The project’s state and national monitoring and evaluation teams routinely checked the quality of these data. Discrepancies in notification data extracted from *Ni-kshay* for our analyses were cross-checked and cleaned using the records at the project sites.

### Statistical analysis

We summarized patient and provider data using frequencies and proportions. We used Excel 2016 (Microsoft, Redmond, United States of America) and Stata 13 (StataCorp. LP, College Station, USA) to analyse the data.

### Ethical considerations

FIND undertook the project with approval from and in collaboration with the national tuberculosis programme. The project implemented approved interventions that were part of the standard tuberculosis care in India. We undertook a desk-based review and analysis of anonymized data available from *Ni-kshay*. Therefore, we did not require a separate ethical clearance.

## Results

### Characteristics

The number of tuberculosis cases notified by the private sector in the project districts in 2017 was 44 695 (Table 1), which increased to 70 353 in 2018 and 106 404 in 2019. In 2019, the mean age of cases notified was 40 years (standard deviation: 18; Table 1). Of the cases notified in 2019 for whom data were available, 5.2% (5511/106 315) were paediatric cases (age 0–14 years), 58.9% (62 633/106 349) were males and 67.3% (68 559/101 878) were pulmonary tuberculosis cases. The percentage of extra-pulmonary cases notified more than doubled between 2017 and 2019 from 16.2% (5971/36 748) to 32.7% (33 319/101 878). As regards diagnosis, 31.1% (32 797/105 356) of cases notified were confirmed microbiologically in 2019. Of the 50 430 cases diagnosed microbiologically in 2018 and 2019, 1310 (2.6%) were rifampicin-resistant. HIV status was known in 78.1% (83 150/106 404) of cases in 2019 and of these cases, 0.8% (671/83 150) were HIV positive. The proportion of missing data on these variables among tuberculosis cases notified to *Ni-kshay* from the project districts decreased considerably from 2017 to 2019.

**Table 1:** Characteristics of tuberculosis cases notified by the private sector, India, 2017–2019

Variable	2017			2018			2019	
	No. (%) cases with data available)	% total cases (*n* = 44 695)		No. (%) cases with data available)	% total cases (*n* = 70 353)		No. (%) cases with data available)	% total cases (*n* = 106 404)
**Age in years, mean (SD)**	40 (18)			39 (18)			40 (18)	
**Age group, in years **								
0–14	1 945 (4.5)	4.4		4 018 (5.8)	5.7		5 511 (5.2)	5.2
15–24	7 785 (17.9)	17.4		12 607 (18.2)	17.9		20 010 (18.8)	18.8
25–34	8 239 (18.9)	18.4		12 897(18.7)	18.3		20 664 (19.4)	19.4
35–44	7 372 (16.9)	16.5		11 426 (16.5)	16.2		17 081(16.1)	16.1
45–54	7 537 (17.3)	16.9		11 406 (16.5)	16.2		16 804 (15.8)	15.8
55–64	5 782 (13.3)	12.9		9 121 (13.2)	13.0		14 192 (13.3)	13.3
≥ 65	4 843 (11.1)	10.8		7 611 (11.0)	10.8		12 053 (11.3)	11.3
Subtotal	43 503 (100.0)	97.3		69 086 (100.0)	98.2		106 315 (100.0)	99.9
Data not available	1 192 (NA)	2.7		1 267 (NA)	1.8		89 (NA)	0.1
**Gender**								
Female	15 889 (36.5)	35.5		26 850 (38.9)	38.2		43 629 (41.0)	41.0
Male	27 600 (63.4)	61.8		42 184 (61.0)	60.0		62 633 (58.9)	58.9
Third gender^a^	14 (0.0)	0.03		66 (0.1)	0.1		87 (0.1)	0.1
Subtotal	43 503 (100.0)	97.3		69 100 (100.0)	98.2		106 349 (100.0)	99.9
Data not available	1 192 (NA)	2.7		1 253 (NA)	1.8		55 (NA)	0.1
**Site of tuberculosis**								
Pulmonary	30 777 (83.8)	69.8		46 296 (76.5)	65.8		68 559 (67.3)	64.4
Extra-pulmonary	5 971 (16.2)	13.4		14 214 (23.5)	20.2		33 319 (32.7)	31.3
Subtotal	36 748 (100.0)	82.2		60 510 (100.0)	86.0		101 878 (100.0)	95.7
Data not available	7 947 (NA)	17.8		9 843 (NA)	14.0		4 526 (NA)	4.3
**HIV status of notified tuberculosis patients**								
HIV positive	145 (1.6)	0.3		845 (2.4)	1.2		671 (0.8)	0.6
HIV negative	8 819 (98.4)	19.7		34 453 (97.6)	49.0		82 479 (99.2)	77.5
Subtotal	8 964 (100.0)	20.1		35 298 (100.0)	50.2		83 150 (100.0)	78.1
Data not available	35 731 (NA)	79.9		35 055 (NA)	49.8		23 254 (NA)	21.9
**Basis of diagnosis**								
Microbiological	12 441 (54.0)	27.8		17 633 (32.5)	25.1		32 797 (31.1)	30.8
Clinical	10 599 (46.0)	23.7		36 560 (67.5)	52.0		72 559 (68.9)	68.2
Subtotal	23 040 (100.0)	51.5		54 193 (100.0)	77.0		105 356 (100.0)	99.0
Data not available	21 655 (NA)	48.5		16 160 (NA)	23.0		1 048 (NA)	1.0

SD: standard deviation; NA: not applicable; HIV: human immunodeficiency virus.

^a^ Individuals who do not identify themselves as female or male.

Note: Data are from districts in areas where FIND implemented the Joint Effort for Elimination of Tuberculosis project.

### Case notifications

Overall, the number of tuberculosis cases notified increased by 138.1% (61 709/44 695) from 2017 to 2019, and the case notification rate increased by 122.1% from 19.6 to 43.5 cases per 100 000 population during this period (Table 2). In districts implementing the patient-provider support agency model, case notifications increased by 236.4%, from 12 920 in 2017 to 43 459 in 2019, while they increased by 98.1% in the patient-provider support agency lite districts from 31 775 to 62 945. The change in the case notification rate followed a similar pattern, being considerably higher in patient-provider support agency districts (229.9%) than patient-provider support agency lite (82.8%) districts. In the states with districts implementing both patient-provider support agency and patient-provider support agency lite, the change in notification rate from 2017 to 2019 was the highest in Telangana (251.2%), followed by Punjab (177.9%); the lowest change was in Andhra Pradesh (67.2%). Himachal Pradesh and Chandigarh were not considered here as they only implemented the lite model and their case notifications were substantially lower than other areas, hence potentially skewing the data (Table 3).

**Table 2:** Tuberculosis cases notified by the private sector, by model implemented, India, 2017–2019

Model type	Tuberculosis case notifications					Population, millions			Tuberculosis case notification per 100 000 population		
	2017	2018	2019	% change 2017–2019		2017^a^	2019^b^		2017	2019	% change 2017–2019
	No.	No.	No.								
Patient-provider support agency	12 920	23 274	43 459	236.4		42.7	43.6		30.2	99.7	229.9
Patient-provider support agency lite	31 775	47 079	62 945	98.1		185.4	201.0		17.1	31.3	82.8
**Total**	**44 695**	**70 353**	**106 404**	**138.1**		**228.1**	**244.5**		**19.6**	**43.5**	**122.1**

^a^ Population data for 2017 were taken from India annual tuberculosis report 2018.^16^

^b^ Population data for 2019 were taken from national tuberculosis programme’s estimates (personal communication, Central Tuberculosis Division, India).

Notes: Data are from districts in areas where FIND implemented the Joint Effort for Elimination of Tuberculosis project. The project did not start until 2018; the data for 2017 were taken for the same districts included in the project. The patient-provider support agency model had extensive human resources whereas the patient-provider support agency lite model had limited human resources. Inconsistencies arise in some values due to rounding of population figures.

**Table 3:** Tuberculosis cases notified by the private sector, by state and model, India, 2017 and 2019

State and model^a^	Districts or cities covered, no.	Case notifications, no.				Population, millions			Case notification rate/100 000			Change^d^ in rate 2017 to 2019, %
		2017	2018	2019		2017^b^	2019^c^		2017	2019		
**Andhra Pradesh**												
Full	1	1 626	2 014	4 039		4.5	4.5		36.4	89.1		145.1
Lite	12	13 555	23 359	21 696		47.0	47.7		28.8	45.5		57.9
Subtotal	13	15 181	25 373	25 735		51.5	52.2		29.5	49.3		67.2
**Karnataka**												
Full	3	2 842	3 696	8 898		11.4	11.7		24.9	76.0		205.5
Lite	16	5 614	7 977	9 641		38.0	36.9		14.8	26.2		77.3
Subtotal	19	8 456	11 673	18 539		49.5	48.6		17.1	38.2		123.3
**Telangana**												
Full	4	969	3 553	9 962		9.7	9.8		10.0	101.2		913.7
Lite	17	5 443	4 916	12 698		17.1	17.1		31.9	74.2		132.9
Subtotal	21	6 412	8 469	22 660		26.8	26.9		23.9	84.1		251.2
**Punjab**												
Full	2	2 549	6 876	10 412		6.1	6.2		41.9	167.5		299.6
Lite	6	2 578	2 834	4 152		10.6	10.8		24.3	38.3		57.6
Subtotal	8	5 127	9 710	14 564		16.7	17.1		30.7	85.3		177.9
**West Bengal**												
Full	11	4 934	7 135	10 148		9.9	10.1		49.8	100.4		101.6
Lite	18	4 254	7 332	13 155		68.5	84.3		6.2	15.6		151.4
Subtotal	29	9 188	14 467	23 303		78.4	94.4		11.7	24.7		110.7
**Himachal Pradesh**												
Lite	4	222	476	1 097		4.1	4.2		5.4	26.0		384.7
**Chandigarh**												
Lite	1	109	185	506		1.1	1.2		9.6	43.6		353.7
**Total**	**95**	**44 695**	**70 353**	**106 404**		**228.1**	**244.5**		**19.6**	**43.5**		**122.1**

^a^ The model is a patient–provider support agency. The full patient-provider support agency model had extensive human resources whereas the patient-provider support agency lite model had limited human resources.

^b^ Population data for 2017 were taken from India annual tuberculosis report 2018.^16^

^c^ Population data for 2019 were taken from national tuberculosis programme’s estimates (personal communication, Central Tuberculosis Division, India).

^d^ Because of small numbers for some models in certain states, some changes presented may be skewed.

Notes: Data are from districts in areas where FIND implemented the Joint Effort for Elimination of Tuberculosis project. The project did not start until 2018; the data for 2017 were taken for the same districts included in the project. Inconsistencies arise in some values due to rounding of population figures.

### Private providers

The number of private providers who submitted tuberculosis notifications increased by 227.1% (6613/2912) from 2017 to 2019, with increases for all types of private facility including health facilities, pharmacies and laboratories (Table 4). In the patient-provider support agency districts, the number of private sector notifiers quadrupled from 809 to 3435 from 2017 to 2019, while in patient-provider support agency lite districts, the number of notifiers almost tripled from 2103 in 2017 to 6090 in 2019.

**Table 4:** Type of private sector notifiers by model implemented and facility type, India, 2017–2019

Model and facility type	Notifiers, no. (%)		
	2017 (*n* = 2912)	2018 (*n* = 6178)	2019(*n* = 9525)
**Model type**			
Patient-provider support agency	809 (27.8)	1836 (29.7)	3435 (36.1)
Patient-provider support agency lite	2103 (72.2)	4342 (70.3)	6090 (63.9)
**Private facility type**			
Pharmacy	65 (2.2)	1098 (17.8)	1253 (13.2)
Health facility	2710 (93.1)	4286 (69.4)	6562 (68.9)
Laboratory	137 (4.7)	794 (12.9)	1710 (18.0)

Notes: Data are from districts in areas where FIND implemented the Joint Effort for Elimination of Tuberculosis project. The project did not start until 2018; the data for 2017 were taken for the same districts included in the project. The patient-provider support agency model had extensive human resources whereas the patient-provider support agency lite model had limited human resources.

### Microbiological validation

The number of notified microbiologically confirmed pulmonary tuberculosis cases more than doubled from 2017 to 2019 from 10 780 to 25 384, a 135.5% increase, as did the number of notified pulmonary tuberculosis cases, from 31 358 to 70 318, a 124.2% increase (Table 5). The number of notified extra-pulmonary tuberculosis cases increased by 412.0%, from 6142 in 2017 to 31 446 in 2019, and the number of notified microbiologically confirmed extra-pulmonary cases rose from 1477 to 4096, a 177.3% increase. However, the proportion of microbiologically confirmed extra-pulmonary cases decreased from 28.9% (1477/5114) in 2017 (data were not available on 16.7%, 1028/6142, of cases) to 13.3% (4096/30 810) in 2019.

**Table 5:** Tuberculosis cases notified by the private sector, by type of tuberculosis, project model and method of diagnosis, India, 2017–2019

Variable	2017			2018			2019	
	No. (%) cases with data available)	% total cases		No. (%) cases with data available)	% total cases		No. (%) cases with data available)	% total cases
**Pulmonary tuberculosis^a^**								
Microbiological	10 780 (58.3)	34.4		14 040 (37.0)	30.2		25 384 (36.8)	36.0
Clinical	7 698 (41.7)	24.5		23 883 (63.0)	51.3		43 617 (63.2)	62.0
Subtotal	18 478 (100.0)	58.9		37 923 (100.0)	81.5		69 001 (100.0)	98.0
Missing	12 880 (NA)	41.1		8 593 (NA)	18.5		1 380 (NA)	2.0
**Total**	**31 358 (NA)**	**100.0**		**46 516 (NA)**	**100.0**		**70 381 (NA)**	**100.0**
**Extra-pulmonary tuberculosis^a^**								
Microbiological	1 477 (28.9)	24.0		1 828 (14.3)	13.6		4 096 (13.3)	13.0
Clinical	3 637 (71.1)	59.2		10 980 (85.7)	81.6		26 714 (86.7)	85.0
Subtotal	5 114 (100.0)	83.3		12 808 (100.0)	95.2		30 810 (100.0)	98.0
Missing	1 028 (NA)	16.7		651 (NA)	4.8		636 (NA)	2.0
**Total**	**6 142 (NA)**	**100.0**		**13 459 (NA)**	**100.0**		**31 446 (NA)**	**100.0**
**Patient-provider support agency**								
Microbiological	3 425 (44.8)	26.5		8 144 (44.8)	35.0		17 869 (41.5)	41.1
Clinical	4 214 (55.2)	32.6		10 037 (55.2)	43.1		25 208 (58.5)	58.0
Subtotal	7 639 (100.0)	59.1		18 181 (100.0)	78.1		43 077 (100.0)	99.1
Missing	5 281 (NA)	40.9		5 093 (NA)	21.9		382 (NA)	0.9
**Total**	**12 920 (NA)**	**100.0**		**23 274 (NA)**	**100.0**		**43 459 (NA)**	**100.0**
**Patient-provider support agency lite**								
Microbiological	9 016 (58.5)	28.4		9 489 (26.3)	20.2		14 928 (24.0)	23.7
Clinical	6 385 (41.5)	20.1		26 523 (73.7)	56.3		47 351 (76.0)	75.2
Subtotal	154 01 (100.0)	48.5		36 012 (100.0)	76.5		62 279 (100.0)	98.9
Missing	16 374 (NA)	51.5		11 067 (NA)	23.5		666 (NA)	1.1
**Total**	**31 775 (NA)**	**100.0**		**47 079 (NA)**	**100.0**		**62 945 (NA)**	**100.0**

NA: not applicable.

^a^ There were 7195 cases in 2017, 10 378 in 2018 and 4577 in 2019 that could not be classified as pulmonary or extra-pulmonary in the *Ni-kshay* surveillance system; these cases are excluded from the analysis.

Notes: Data are from districts in areas where FIND implemented the Joint Effort for Elimination of Tuberculosis project. The project did not start until 2018; the data for 2017 were taken for the same districts included in the project. The patient-provider support agency model had extensive human resources whereas the patient-provider support agency lite model had limited human resources.

In the patient-provider support agency districts, of cases for whom data were available, microbiologically confirmed tuberculosis cases accounted for 41.5% (17 869/43 077) of cases in 2019 compared with 44.8% (3425/7639) in 2017 (Table 5).

The proportion of microbiologically diagnosed cases in all states was between 20.5% (5264/25 717) and 53.4% (270/506).

### Project and non-project districts 

The case notification rate increased more substantially in project districts than non-project districts in all states between 2017 and 2019 (Table 6). Overall, districts where the project was implemented showed a 150.3% increase in the case notification rate from 2017 to 2019 (from 16.8 to 41.9 cases per 100 000 population), while this increase was 89.8% in non-project districts (from 6.1 to 11.6 cases per 100 000 population). 

**Table 6:** Tuberculosis cases notified by the private sector, by state, India, 2017 and 2019

State and type of district	2017 notifications				2019 notifications			Change in rate 2017 to 2019, %^c^
	Population (millions)^a^	No.	Case notification rate/100 000		Population (millions)^b^	No.	Case notification rate/100 000	
**Himachal Pradesh**								
Project	4.1	222	5.4		4.2	1 097	26.0	384.7
Non-project	3.1	248	7.9		3.2	522	16.3	106.4
Subtotal	7.2	470	6.5		7.4	1 619	21.8	237.9
**Karnataka**								
Project	49.5	8 456	17.1		48.6	18 539	38.2	123.3
Non-project	16.6	1 812	10.9		17.0	2 331	13.7	25.5
Subtotal	66.0	10 268	15.5		65.6	20 870	31.8	104.8
**Punjab**								
Project	16.7	5 127	30.7		17.1	14 564	85.3	177.9
Non-project	13.0	821	6.3		13.3	584	4.4	−30.4
Subtotal	29.7	5 948	20.0		30.3	15 148	49.9	149.1
**Telangana**								
Project	26.8	6 412	23.9		26.9	22 660	84.1	251.2
Non-project	10.8	108	1.0		11.0	1 871	17.1	1602.7
Subtotal	37.6	6 520	17.4		37.9	24 531	64.7	272.7
**West Bengal**								
Project	78.4	9 188	11.7		94.4	23 303	24.7	110.7
Non-project	21.3	983	4.6		21.8	2 399	11.0	138.4
Subtotal	99.7	10 171	10.2		116.2	25 702	22.1	116.9
**Total**								
Project	175.5	29 405	16.8		191.2	80 163	41.9	150.3
Non-project	64.8	3 972	6.1		66.2	7 707	11.6	89.8
**Overall total**	**240.3**	**33 377**	**13.9**		**257.4**	**87 870**	**34.1**	**145.8**

^a^ Population data for 2017 were taken from India annual tuberculosis report 2018.^16^

^b^ Population data for 2019 were taken from national tuberculosis programme’s estimates (personal communication, Central Tuberculosis Division, India).

^c^ Because of small numbers for type of district, change in rate between 2017 and 2019 presented may be skewed.

Notes: Data are from districts in areas where FIND implemented the Joint Effort for Elimination of Tuberculosis project. The project did not start until 2018; the data for 2017 were taken for the same districts included or not included in the project. Inconsistencies arise in some values due to rounding of population figures.

## Discussion

We found a rapid and substantial increase in tuberculosis case notifications, private provider notifiers and microbiological confirmation of cases in districts between 2017 and 2019. In addition, the case notification rate was higher in districts where the Joint Effort for Elimination of Tuberculosis project had been implemented by FIND compared with districts where it was not implemented.

Attempts to improve notifications from the private sector over the past two decades in India have resulted in only small increases which are not enough to achieve the country’s tuberculosis elimination targets.^2^ The findings of our study have implications for several documented challenges in engaging the private sector.^2^^,^^9^^,^^20^^,^^21^ Our findings show that substantial improvement in private provider engagement and tuberculosis case notification can be achieved by using previous successful models and the existing systems put in place by the national tuberculosis programme. The patient-provider support agency model is a scale up of the private-provider interface agency model implemented in Mumbai (Maharashtra), Patna (Bihar) and Mehsana (Gujarat), which was a cost-effective initiative to engage private providers.^22^^,^^23^ The 58% increase seen in notifications in Mumbai from 2013 to 2017 was attributed to the private-provider interface agency model.^24^ Our comparison of project and non-project districts showed a marked increase in notification rates in just 2 years of implementation of the project. Indeed, the private sector case notification rate from non-project districts also increased over the same period, which was expected as a result of expanded efforts by the national tuberculosis programme. However, we saw a considerably greater increase in notification rates in the project districts compared with non-project districts.

Accurate diagnosis of tuberculosis, including drug resistance, and the need to increase the proportion of bacteriologically confirmed cases is increasingly emphasized globally. Hence, scaling up access to sensitive diagnostics, including drug susceptibility testing, is a national priority. Our data show a substantial increase in the proportion of microbiologically confirmed cases in patient-provider support agency districts during the study period. This finding reflects the project’s focus on enhancing microbiological confirmation through the patient-provider support agency model. Furthermore, the project actively engaged several providers other than lung health specialists who dealt with extra-pulmonary tuberculosis cases, such as paediatricians, orthopaedic physicians and surgeons. This strategy could partially explain the increase in the number of microbiologically confirmed extra-pulmonary cases which would have gone undiagnosed in the absence of such efforts. The proportion of microbiologically diagnosed cases in all states was between 20.5% and 53.4%. Some^12^^,^^24^ but not all^25^ studies on programmes including the private sector in a few Indian states reported the percentage of microbiologically confirmed cases in this range (35–60%).

Our results reflect several successful partnerships and measures used to engage the private health sector which were scaled up by the project. First, the project’s goals and strategies were in alignment with national priorities, and ranged from extensive mapping and engagement of private providers to their consistent and structured follow-up to ensure end-to-end involvement. Second, compared with previous referral-based engagement models that relied on altruistic motivation and/or incentives, the project intervention offered tangible, non-monetary incentives to private providers and made efforts to protect the business interests of the private sector by providing the option to retain their patients rather than transferring them to the public care system. Past referral-based public–private mix models resulted in increases in overall notification rates of 12–98%, and 2–26% of new case notifications in the private sector.^8^ In contrast, the patient-provider support agency model resulted in greater improvements in case notification rates in the project districts (69.0–250.0% increase). The activities in the patient-provider support agency model that supported its success included: systematic mapping and strategic engagement of private providers offering tuberculosis care; free sample collection, quality checks of samples, transportation of samples and reports, and links to free molecular testing near the private provider; support to notify cases; linking of patients to free treatment; support for treatment adherence through dedicated resources; timely feedback and liaison with stakeholders; and facilitating of incentives to providers and patients as per the national tuberculosis programme network. Third, FIND deployed the intervention through intermediary agencies that had already facilitated effective interventions to engage the private sector in the project states.^14^ These agencies used their own successful strategies which included focus on targeted communication, innovative ideas to accommodate regional variations, simplified processes to involve providers, and partnerships to maintain trust of the engaged providers.^15^ Fourth, the government’s existing web portal *Ni-kshay* was used to capture the data from the project, which avoided duplication of data as no additional information management platform was used. Fifth, the project was implemented in close coordination with the national tuberculosis programme at national, state and district levels.

A few barriers to implementation of the project need to be mentioned. First, the initiative relied heavily on the national tuberculosis programme for supply of services. Delays in implementation of services, such as cartridge-based nucleic acid amplification assay tests, and increased testing loads at public sector laboratories affected turnaround time of test results. The project supported states to have optimal stocks of cartridges through timely forecasts and context-adapted remedial measures to mitigate cartridge shortages on the ground. Furthermore, the project optimized the network of available cartridge-based nucleic acid amplification assay machines. In addition, many stakeholder meetings were conducted between the national tuberculosis programme officials, key private hospitals with in-house testing facilities and partners. Consequently, the project succeeded in using other resources, such as the laboratory technician scheme, public–private mix, drug susceptibility testing schemes, and provision of cartridge-based nucleic acid amplification assay testing services free-of-charge to the private sector so patients could be tested within the private sector without charge. These measures resulted in accelerated access to improved diagnostics. Second, patients and providers had concerns about the quality and efficacy of free antituberculosis drugs available in the public sector. These concerns resulted in low uptake of these drugs by patients approaching the private sector. This challenge was mitigated by evidence-based sensitization of patients and providers on the high quality of the free antituberculosis drugs. Third, the provision of patient and provider incentives were delayed at times. This issue was mitigated through continuous and rigorous advocacy with the state and national tuberculosis programme officials to streamline the process of delivery of these incentives.

Our analysis has a few limitations. First, our data were limited to private providers mapped under the project. Hence, our results may not represent all private sector notifications in the project districts. However, the proportion of providers outside the project network is likely to be negligible because of the rigorous provider mapping that was done. Second, our comparison of project and non-project districts reflects the change the project brought about in the notification rate from 2017 to 2019. However, factors such as the proportion of urban and rural populations in these states, the number of health care providers per district and the facilities where health care is accessed by the population were not accounted for, which limits direct comparison of project and non-project districts. Third, the proportion of notified cases diagnosed microbiologically for whom data were available decreased from 54.0% (12 441/23 040) in 2017 to 31.1% (32 797/105 356) in 2019. This decrease can be attributed to: (i) missing data on nearly half of the cases in 2017 compared with only 1.0% (1048/106 404) in 2019 in the *Ni-kshay*; (ii) underreporting of microbiological confirmation as the project was unable to capture the status of samples analysed in private laboratories because of lack of access before diagnosis; (iii) the fact that the *Ni-kshay* registers information based on diagnosis from the results of the first test for tuberculosis conducted and misses subsequent tests; and (iv) the large increase in notifications between 2017 and 2019, which concurs with the inverse association documented between notification numbers and proportion of microbiologically confirmed cases in high burden countries in 2018–2019.^1^ However, the number of microbiologically confirmed cases more than doubled from 2017 to 2019 through efforts of the project. The proportion increased in pulmonary tuberculosis cases (which make up most of the cases) in patient-provider support agency districts where a great effort was made to facilitate microbiological confirmation of tuberculosis. Fourth, we did not assess factors related to the performance of the project through a comparison of intervention and control districts. While observer-induced factors may partially explain our findings, we attempted to mitigate such factors by comparing the results from intervention districts with non-intervention districts within states. Fifth, most variables had missing data. However, missing data decreased substantially from 2017 to 2019. In addition to the systematic efforts of the national programme, this improvement in data quality can be attributed to factors such as facilitation of microbiological confirmation, and improved recording and reporting structures which were emphasized by the project.

The findings of our study are in alignment with experiences shared by consortium partners implementing the project in other states of India. The project findings may be generalizable to areas with a high tuberculosis burden and with a sizable private sector presence in these areas.

In conclusion, the national strategic plan, 2017–2025, indicates that involving the private sector may result in doubling or tripling case notification rates. The findings of our study validate this assumption. The patient-provider support agency model demonstrates that the potential of the private sector can be used to scale up tuberculosis reporting through key actions to successfully engage this sector. India’s national tuberculosis programme has set a target for 2020–2023 that 56% of total case notifications should come from the private sector. The substantial increase achieved by the project in the six states presented here is relevant to this target. The initiative is being sustained through domestic funds in all patient-provider support agency districts to maintain the gains made. Overall, the project is a highly sustainable model of public–private cooperation that could inform the national tuberculosis programme about the feasibility and operational aspects of future schemes to engage the private sector and move from a project to a programme model.
